# Tinnitus, temporomandibular disorders and muscle activity: what's the connection?

**DOI:** 10.1590/1807-3107bor-2026.vol40.037

**Published:** 2026-06-22

**Authors:** Elif Çoban, Berkan Altay, Mehmet Çağatay Ulucan, Selver Suna BaŜak, Berceste Güler, Merve Akdeniz Leblebiciler, Vural Kavuncu

**Affiliations:** (a) Kırıkkale University, Department of Oral and Maxillofacial Surgery, Türkiye; (b) Prosthodontist, Eskişehir Oral and Dental Health Hospital, Eskişehir, Türkiye.; (c) Kahramanmaraş Sütçü İmam University, Department of Prosthodontics, Kahramanmaraş, Türkiye; (d) Kütahya Health Sciences University, Department of Periodontology, Kütahya, Türkiye.; (e) Kütahya Health Sciences University, Department of Physical Medicine and Rehabilitation, Kütahya, Türkiye.

**Keywords:** Tinnitus, Splints, Temporomandibular Joint

## Abstract

This study was aimed at comparatively evaluating the relationship between temporomandibular disorder (TMD) and subjective tinnitus, as well as the effects of TMD treatment on masticatory muscle activity, tinnitus symptoms, and patient perception. In this prospective cohort study, patients diagnosed with both TMD and tinnitus were divided into groups with or without bruxism. The inclusion criteria were confirmed diagnosis of TMD and tinnitus. The exclusion criteria were history of prior TMD treatment, neurological disorders, or systemic diseases affecting muscle function. The primary outcome measures were electromyographic (EMG) assessments of masseter muscle maximum voluntary contraction (MVC) and the percentage of overlapping coefficient (%POC), determined via surface EMG (sEMG). The secondary outcomes included changes in tinnitus symptoms, assessed with the Tinnitus Handicap Inventory (THI), and pain levels, evaluated with the Visual Analog Scale (VAS). A total of 70 eligible participants were evenly distributed between groups. Tinnitus symptoms resolved in 35.7% of patients and did not significantly differ between groups (p > 0.05). Masseter MVC values decreased numerically but non–significantly after treatment (p > 0.05). In contrast, significant post–treatment improvements were observed in %POC, VAS, and THI scores (p < 0.05). Pre– and post–treatment comparisons of MVC, %POC, VAS, and THI revealed no significant intergroup differences (p > 0.05). TMD treatment appeared to effectively decrease subjective tinnitus symptoms and improve quality of life. Our findings suggest that TMD management might play an important role in tinnitus treatment and indicate that tinnitus is more associated with underlying internal disorders than with masticatory muscle activity directly influenced by bruxism.

## Introduction

Tinnitus is defined as the perception of sounds in the ears or inside the head in the absence of any external acoustic stimulus.^1^ Commonly experienced as a ringing sensation, tinnitus markedly diminishes individuals’ quality of life and predisposes them to various psychological problems.^2^ Unlike normal tinnitus, which can be experienced by many individuals, pathological tinnitus is characterized by episodes persisting for > 5 minutes and occurring over the course of > 1 week.^3^

The pathophysiology of tinnitus remains incompletely understood, but temporomandibular disorders (TMDs) have been identified as risk factors for its onset and exacerbation.^4^ This association might arise from the anatomical and functional interconnections among the temporomandibular joint (TMJ), masticatory muscles, and auditory system.^5,6^ Microtrauma, such as bruxism caused by repetitive oral activities, is another risk factor that contributes to the onset and persistence of TMD. Increased masticatory muscle activity due to bruxism has been associated with tinnitus symptoms.^7^ In particular, increased tension in the masticatory muscles may place mechanical stress on the discomalleolar ligament, which forms a direct anatomical connection between the TMJ and the middle ear, thereby producing otologic manifestations.^8^ Furthermore, hyperactivity of the masticatory muscles may interfere with the coordinated function of the tensor tympani and tensor veli palatini muscles, thereby altering the position of the tympanic membrane and the malleus.^8,9^ These biomechanical disruptions within the stomatognathic system can ultimately contribute to the perception of tinnitus. Consequently, TMD treatment is important for substantially improving tinnitus management. Effective treatment of TMD in patients with concurrent tinnitus has been shown to significantly decrease or even eliminate tinnitus severity.^4^

Surface electromyography (sEMG) of the masticatory muscles, a valuable tool to assess disease progression and treatment efficacy,^10^ enables monitoring of changes in the muscle patterns of the stomatognathic system.^11^ Ferrario et al. have introduced a standardized sEMG protocol with EMG signals and indices for masticatory muscles.^12^ sEMG analyses of the masticatory muscles have been used to evaluate the effects of splint therapy on masticatory muscle activity.^10^ Therefore, investigating the relationships between changes in masticatory muscle activity and alterations in TMD and tinnitus symptoms may provide crucial insights.

Despite increasing evidence of the relationship between TMD and tinnitus, the exact mechanism linking masticatory muscle activity to tinnitus perception remains unclear. Previous studies have focused primarily on clinical findings rather than objective neuromuscular assessments. To address this research gap, this study was aimed at investigating whether decreases in masseter muscle activity after TMD treatment might correlate with improvement in tinnitus symptoms. The primary objective was to explore this correlation, whereas secondary aims included evaluating alterations in masseter activity and tinnitus scores before and after treatment.

## Methods

The study, conducted at the Department of Oral and Maxillofacial Surgery, Faculty of Dentistry, Kütahya Health Sciences University, included patients with TMD and subjective tinnitus. The study was designed as a prospective cohort comparative trial. Ethical approval for the study was obtained from the Kütahya Health Sciences University Clinical Research Ethics Committee, under decision number 2021–09/07. The study was conducted in accordance with the guidelines of the Helsinki Declaration of Human Rights, and written consent was obtained from each patient.

### Sample Size

The sample size for this study was determined with appropriate software, according to a previously established effect size.^13^ A total of 70 participants were enrolled in the study, and the effect size was calculated to be 0.08 for significance, α = 0.05, and 80% power analysis.

### Study Groups

Patients were consecutively recruited and divided into two groups according to the presence of bruxism. Group I consisted of individuals with TMD and subjective tinnitus (n = 35), and group II included patients with TMD, subjective tinnitus, and bruxism (n = 35). The inclusion criteria were individuals 18 years or older presenting with pathologic tinnitus, subjective tinnitus, or concerns of tinnitus at the Ear, Nose, and Throat Department, and who had Wilkes stage 1–2 TMD. Subjective tinnitus was defined as the perception of sound in the absence of an external acoustic stimulus and without any measurable objective sound during audiological evaluation. Unlike normal tinnitus, which can occasionally occur in healthy individuals and resolve spontaneously, subjective tinnitus is considered pathological when episodes persist for more than 5 minutes and last for more than 1 week.

The exclusion criteria included patients with normal, objective, and/or audiological, neurological, metabolic, or pathologically induced tinnitus; patients with Wilkes stage 3–4–5 TMD; patients with hearing loss, ear pathology, ototoxic drug use, Meniere's disease, history of traumatic cervical spine injury, or depression diagnosed by a psychologist; and patients who had undergone TMD treatment within the preceding 2 months.

### Study design

Demographic data including age, sex, systemic disease, and medication and trauma history were collected. Patients were evaluated for maxillofacial pathologies, TMD, bruxism, and tinnitus. TMJ examination was performed clinically and radiologically, and the Wilkes stage was determined. Bruxism was identified as probable bruxism, determined through a combination of self–reported habits (diurnal or nocturnal clenching and grinding) and clinical indicators such as tooth wear, linea alba, buccal mucosa indentations, morning jaw fatigue, and masseter hypertrophy on palpation. Because polysomnographic evaluation was not available, the diagnosis relied on clinical and anamnestic findings, which were assessed by a calibrated oral surgeon using a standardized checklist to improve diagnostic reliability. Each patient answered a questionnaire pertaining to symptoms before and 6 months after treatment, and the masseter muscles were evaluated with sEMG. A stabilization splint was provided to each patient. Patients were asked to wear the stabilization splint every night. Patients were taught how to apply and remove the splint correctly. Clinical controls were performed at 1 week and thereafter monthly for 6 months, to ensure compliance and adjust occlusal contacts. Although no splint–free control group was included, because of ethical concerns regarding withholding standard treatment from symptomatic TMD patients, intra– and inter–group comparisons were performed to assess the treatment effects. The questionnaires administered to the patients before treatment and after treatment were VAS and THI.

**Figure 1:**
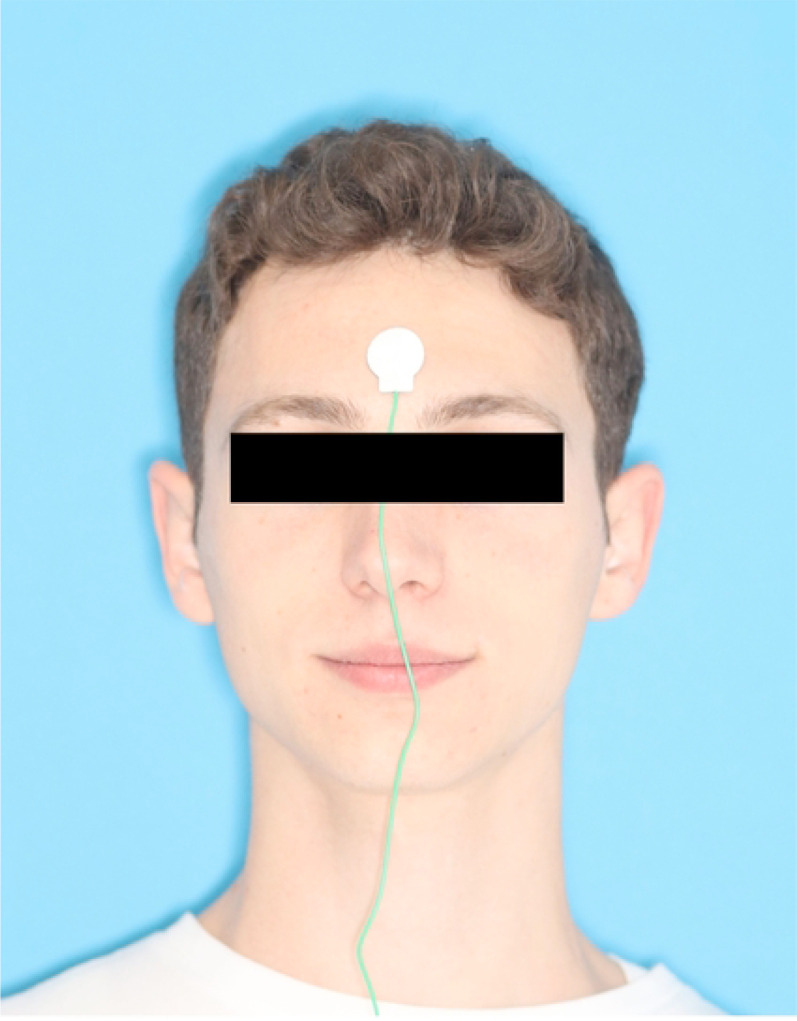
The reference electrode was positioned on the patient's forehead along the midline to minimize noise and ensure stable electrical grounding during masseter muscle recordings.

**Figure 2:**
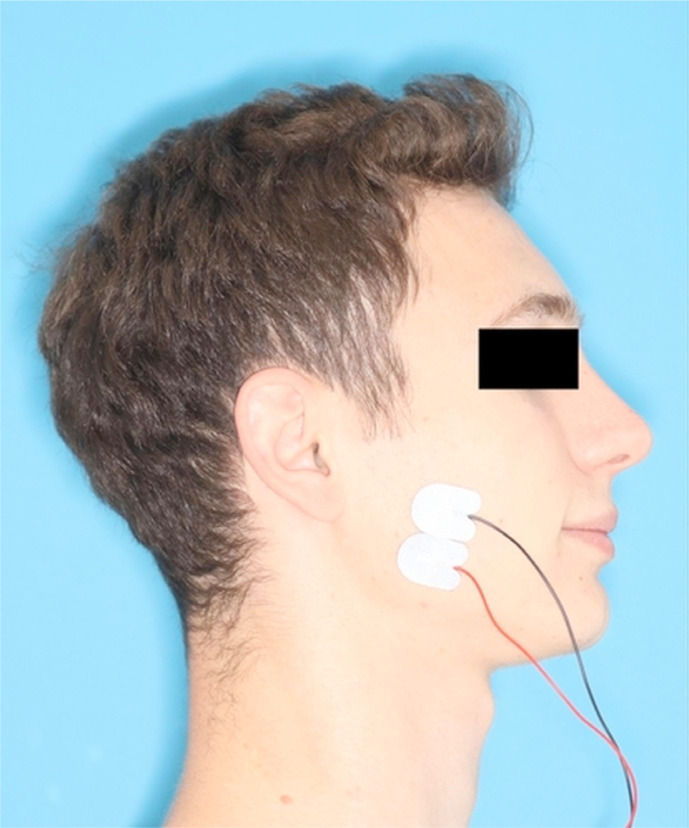
Disposable electrodes were placed on the belly of the masseter muscle along the muscle fibers, maintaining a parallel orientation to ensure accurate detection of muscle activity.

Because the masseter is the most active muscle in mastication, the patients’ masseters were evaluated with sEMG before and after treatment. sEMG was performed by the same physician for all patients, with a protocol described by Ferrario et al.^12^ The analog EMG signal was amplified with a differential amplifier with a high common mode rejection ratio. The signals averaged > 500 ms (Myoquick, Micromed, Italy). The reference electrode was positioned on the front of the head of each patient (Figure 1). Surface electrodes were placed on both masseter muscles (Figure 2). Two 10 mm thick cotton rolls were placed on the mandibular first and second molars of each patient. Each patient was asked to bite down and maintain the same level of contraction for 5 seconds to standardize the sEMG data, and the maximum voluntary contraction (MVC) was recorded. EMG data analysis was performed by selecting the best 3–second period for all tests, which was used for all subsequent analyses. Patients were then asked to clench their teeth as hard as possible in the intercuspal position and to maintain the same level of contraction for 5 seconds, and the MVC was then recorded. For each patient, the EMG potentials of the muscles were analyzed during the MVC tests and are expressed as a percentage of the mean potential recorded during the standardization test with cotton rolls (μV/μV × 100). The sEMG data for the masseter muscles were compared by calculation of the percentage overlapping coefficient (%POC) to assess muscle symmetry as follows: %POC: [1 − (right masseter–left masseter)/(right masseter + left masseter)] x 100.

### Statistical method

Temporal evaluations within each group were analyzed with the Wilcoxon signed rank test, paired sample T–test, and Mann–Whitney U test, depending on the normal distribution of values. For intergroup comparisons, the Wilcoxon signed rank test and chi–square test were used for parametric intragroup evaluations, whereas the Mann–Whitney U test was used for nonparametric comparisons, again depending on the normal distribution of the data. Relationships between variables were assessed with Kendall's tau correlation coefficient. A significance level of 0.05 was used.

## Results

The patients ranged in age from 18 to 70 years. Group I comprised 77.1% women (n = 27) and 22.9% men (n = 8), whereas group II comprised 80% women (n = 28) and 20% men (n = 7). In Group I, 71.4% of patients had Wilkes stage I (n = 25), whereas 28.6% had Wilkes stage II (n = 10). In group II, 88.6% of patients had Wilkes stage I (n = 31), whereas 11.4% had Wilkes stage II (n = 4). However, no statistically significant difference in Wilkes stage was observed between groups (p>0.05). Moreover, no significant differences were found in sEMG or %POC values between groups according to sex, Wilkes stage, trigger points, TMD side, or tinnitus side (p > 0.05; Table 1).

**Table 1:** Comparison of sEMG parameters according to demographic and clinical characteristics.

Variable		T1	T1	T1	T2	T2	T2
		MVC Right (μV/μV×100)	MVC Left (μV/μV×100)	%POC Mean ± SD	MVC Right (μV/μV×100)	MVC Right (μV/μV×100)	%POC
		Mean ± SD	Mean ± SD		Mean ± SD	Mean ± SD	Mean ± SD
Gender							
	Female	115.5 ± 46.9	120.2 ± 56.3	83.8 ± 12.5	109.4 ± 43.9	110.7 ± 48	88.4 ± 8.8
	Male	139 ± 5	151 ± 55.6	90.2 ± 7.9	141.2 ± 45.1	137.7 ± 60.7	88.5 ± 10.9
	p–value	0.61	0.505	0.71	0.24	0.58	0.61
Wilkes stage							
	Stage 1	108.8 ± 41.2	116.3 ± 51.1	85.2 ± 13.1	129.5 ± 47.1	105.4 ± 56	88.4 ± 8.2
	Stage 2	130.6 ± 38.4	122.2 ± 49.9	83.1 ± 10.1	118.4 ± 44.9	119.7 ± 32.8	90.5 ± 6.4
	p–value	0.35	0.99	0.22	0.605	0.91	0.22
Bruxism							
	Yes	127.1 ± 51.4	133.8 ± 66.8	83.7 ± 11.4	129.1 ± 55.8	126.1 ± 65.8	88.2 ± 8.1
	None	111.1 ± 42.8	113.7 ± 50.9	85.8 ± 13.3	103.1 ± 33.4	107.9 ± 38.8	80 ± 9.5
	p–value	0.33	0.182	0.233	0.03*	0.29	0.49
Trigger points							
	Yes	113.6 ± 41.7	114.1 ± 56.7	83.3 ± 14.3	99.8 ± 29	101.9 ± 43.2	90.1 ± 8.5
	None	108.2 ± 37.9	123.9 ± 51.2	88.2 ± 9.3	112.3 ± 43.7	125 ± 61.8	99.1 ± 6.4
	p–value	0.61	0.08	0.36	0.3	0.3	0.43
TMD side							
	Right	118.1 ± 38.1	125.9 ± 46.9	88 ± 10.9	122.5 ± 37.7	105.3 ± 34.1	92.6 ± 7.6
	Left	115.6 ± 43	137.7 ± 67	86.8 ± 6.8	108.7 ± 34.2	139.5 ± 71.7	89.9 ± 7.8
	Both Side	113 ± 46.6	111.6 ± 51.1	83.3 ± 15.8	99.7 ± 41.1	92.7 ± 37.7	87.2 ± 11.1
	p–value	0.59	0.54	0.14	0.27	0.54	0.5
Tinnitus side							
	Right	118.1 ± 35.8	115.5 ± 50.1	84.4 ± 8.9	115.8 ± 35.2	115.8 ± 47.7	88.6 ± 8.4
	Left	101 ± 45.7	118.8 ± 56.5	86.1 ± 10.6	102.3 ± 29	118.8 ± 53.5	91.8 ± 7.9
	Both Side	123.5 ± 56	109 ± 53.6	82.6 ± 14.4	114.2 ± 51.4	99.5 ± 50.8	91.8 ± 10.7
	p–value	0.67	0.92	0.97	0.53	0.93	0.46

MVC: Maximum voluntary contraction; POC: Percentage overlapping coefficient; TMD: Temporomandibular disorder; sEMG: Surface electromyography. T1: Preoperative; T2: Postoperative. No significant differences were found in sEMG or %POC values across subgroups by sex, Wilkes stage, trigger points, TMD side, or tinnitus side.

A moderate, positive correlation was observed between the TMD site and the tinnitus site (r = 0.461, p < 0.001). Similarly, the moderate, positive correlation observed between the onset time of TMD and the onset time of tinnitus (r = 0.398, p < 0.001) indicated that tinnitus symptoms tended to develop in parallel with TMD progression.

Analysis of MVC, %POC, TMD VAS, tinnitus VAS, and THI variables before and after treatment revealed no significant differences between groups (p > 0.05; Table 2). However, within–group comparisons indicated that MVC values of the right and left masseters decreased numerically after treatment, although this decrease did not reach statistical significance (p > 0.05). In contrast, the difference between pre– and post–treatment %POC values was statistically significant, and indicated a decrease in %POC after treatment (p = 0.003, p < 0.05). Furthermore, comparison of pre– and post–treatment scores for TMD VAS, tinnitus VAS, bruxism VAS, and THI revealed significant decreases across all parameters after treatment (p < 0.05), thereby indicating overall symptomatic improvement (p < 0.05; Table 3).

**Table 2:** Comparison of clinical and sEMG parameters between Group I and Group II.

Variables	Group I	Group II	p–value
	(n%)	(n%)	
	Mean + SD	Mean + SD	
T1 TMD VAS	6 (3–10)	6(1–10)	0.255
T2 TMD VAS	2(0–8)	2(0–7)	0.621
T1 Tinnitus VAS	5(2–10)	5(2–10)	0.474
T2 Tinnitus VAS	2(0–8)	2(0–8)	0.590
T1 THI	39.400 ± 27.198	34.685 ± 20.676	0.769
T2 THI	14.057 ± 19.387	12.914 ± 15.441	0.762
T1 Right MVC (μV/μV×100)	111.160 ± 42.824	127.170 ± 51.441	0.333
T1 Left MVC (μV/μV×100)	113.731 ± 50.917	133.845 ± 66.891	0.182
T1 POC%	85.820 ± 13.392	83.74 ± 11.498	0.223
T2 POC%	89.038 ± 9.533	88.298 ± 8.163	0.499
T2 Right MVC (μV/μV×100)	103.137 ± 33.462	129.174 ± 55.849	0.036
T2 Left MVC(μV/μV×100)	107.984 ± 38.858	126.140 ± 65.876	0.299

MVC: Maximum voluntary contraction; POC: Percentage of overlapping coefficient; THI: Tinnitus handicap inventory, TMD: Temporomandibular disorder; T1: Preoperative; T2: Postoperative. Both groups showed a notable postoperative decrease in VAS and THI, along with a mild decrease in MVC values and an increase in %POC, thus indicating improved muscle coordination. No significant differences were observed between groups.

**Table 3:** Comparison of preoperative and postoperative sEMG and clinical parameters

Variables	Preoperative Mean ± SD	Postoperative Mean ± SD	p-value
MVC (μV/μV×100) Right Masseter	119.16 ± 47.67	116.15 ± 47.54	0.402
MVC (μV/μV×100) Left Masseter	123.78 ± 59.87	117.06 ± 54.46	0.063
%POC	84.78 ± 12.43	88.66 ± 8.81	0.003*
TMD VAS	6.17 ± 2.4	2.32 ± 2.1	<0.001*
Bruxism VAS	6.47 ± 2.51	3.62 ± 2.5	<0.001*
Tinnitus VAS	5.34 ± 2.32	3.24 ± 2.03	<0.001*
THI	37.04 ± 24.1	21.8 ± 17.3	<0.001*

MVC: Maximum voluntary contraction; POC: Percentage of overlapping coefficient; THI: Tinnitus handicap inventory; TMD: Temporomandibular disorder.

* p-value < 0.05. Postoperatively, both masseter MVC values showed a slight decrease, whereas %POC values significantly increased (p = 0.003), thus indicating improved muscle coordination. Moreover, all clinical parameters, including TMD–, bruxism–, and tinnitus–related VAS scores, as well as THI scores, demonstrated a significant postoperative decrease reflecting symptomatic improvement after splint therapy.

## Discussion

This prospective cohort study investigated the etiological relationship between subjective tinnitus and TMD. In addition, it evaluated the role of occlusal splints in the management of subjective tinnitus. Stabilization splint therapy may alleviate tinnitus.^13^ Given the anatomical proximity of the TMJ and the ear, along with shared vascular and neural pathways, disorders affecting one system might influence the other.^14,15^ Tinnitus incidence increases with age and is more prevalent in women.^4,16^ In this study, most participants were women, and the mean age was 34 years; however, neither sex nor age showed statistically significant associations with TMD or subjective tinnitus.

The Wilkes classification is widely used for evaluating TMJ pathology, because of its simplicity. ^17^ Most patients in this study were categorized into Wilkes stage I. No significant differences were observed between groups in tinnitus frequency, duration, type, or Wilkes stage. Although tinnitus is typically bilateral, it can also occur unilaterally. In this study, tinnitus was more predominant on the left side, and a moderate, positive correlation was found between the sides affected by TMD and tinnitus. The close onset times of TMD and tinnitus symptoms suggested a functional relationship between conditions, thus supporting the hypothesis that management of TMD might alleviate tinnitus symptoms.^4^

Stabilization splint therapy has been suggested to decrease tinnitus symptoms, particularly in patients with normal hearing and synchronous onset of TMD and tinnitus.^13^ The best outcomes have been observed in younger patients with mild tinnitus.^18,19^ However, TMD treatment has not been reported to change tinnitus in patients with severe tinnitus.^20^ In the present study, 35.7% of patients experienced complete resolution of tinnitus symptoms after splint therapy. This clinically meaningful outcome reinforces the therapeutic potential of TMD management, although the difference between groups was not statistically significant.

Surface electromyography has been used to assess masticatory muscle activity in patients with TMD. Whereas some studies have reported decreased masseter muscle activity after TMD treatment, others have found no significant changes.^21^ We observed no statistically significant differences in MVC values before and after treatment. This finding partially differs from those of Nascimento et al. and Dalewski et al., who have reported decreased MVC after splint therapy.^22–23^ Additionally, masticatory muscle symmetry, as evaluated with the %POC, significantly improved after treatment in both groups, thus demonstrating enhanced muscle balance after stabilization splint therapy. This improvement in muscle symmetry (on the basis of %POC) despite stable MVC values suggests that stabilization splints might not necessarily decrease absolute muscle activation but instead enhance bilateral coordination and functional balance of the masticatory muscles.

Subjective symptoms, assessed with VAS and THI scores, significantly decreased after treatment (p < 0.05), thus demonstrating an overall improvement in both TMD and tinnitus symptoms, regardless of age or sex.^24–25^ However, these changes were not influenced by age or sex. The improvements observed in THI and VAS scores despite unchanged MVC values indicated that perceived symptom relief might not be directly proportional to objective decreases in muscle activity. This finding highlights the complex, multifactorial nature of tinnitus perception, which involves not only neuromuscular but also central auditory and psychological mechanisms.

Several hypotheses have been proposed to explain the relationship between TMD and tinnitus. Anatomical studies suggest that TMJ structures might directly influence the middle ear through connections with the malleus bone, affect tympanic membrane tension, and lead to tinnitus.^5,26^ Others have suggested that increased masticatory muscle tension, bruxism, or TMJ internal derangement might contribute to tinnitus development.^27,28^ However, no clear consensus exists regarding the primary mechanism. In our study, bruxism was the main differentiating factor between groups, yet no significant differences were found in clinical symptoms, sEMG data, or questionnaire responses. Therefore, TMD–associated tinnitus might be more strongly associated with TMJ dysfunction and its anatomic–neural interactions than with muscle hyperactivity alone.

This study has several limitations. The diagnosis of bruxism was based solely on clinical and anamnestic findings without polysomnographic verification, thus potentially limiting diagnostic precision. Additionally, the 6–month follow–up period might have been insufficient to capture long–term neuromuscular adaptations or tinnitus recurrence. A more comprehensive diagnostic protocol and longer observation period might strengthen future evidence of the causal relationship between TMD therapy and tinnitus improvement. Despite these limitations, our findings contribute to the growing body of evidence that tinnitus associated with TMD might be a distinct subtype responsive to TMD management.

A strong relationship between TMD and tinnitus has been reported, and our present findings further support this association. However, the underlying mechanisms remain unclear. Despite the limitations of this study, subjective tinnitus appeared to be more closely associated with TMJ dysfunction and its anatomic connections than with masticatory muscle hyperactivity. Although these findings provide insight into the TMD–tinnitus relationship, confirmation through studies with larger cohorts and longer follow–up periods is needed. Given the multifactorial nature of tinnitus, patients should be assessed within a multidisciplinary framework for accurate diagnosis and effective management. Oral and maxillofacial surgery specialists are encouraged to take an active role in such collaborative approaches and in advancing research on this topic.

## Data Availability

The datasets generated during and/or analyzed during the current study are available from the corresponding author on reasonable request.
